# Walking a tightrope: meaningful encounters in the care for people with dementia and severely challenging behavior

**DOI:** 10.1093/geront/gnag175

**Published:** 2026-08-07

**Authors:** Els Van Wijngaarden, Debby Gerritsen

**Affiliations:** Department of Anesthesiology, Pain and Palliative Medicine, Radboud University Medical Center, Nijmegen, The Netherlands; Department of Primary and Community Care, Radboud University Medical Center, Nijmegen, The Netherlands

**Keywords:** Attunement, Interaction, Phenomenology, Shadowing, Observations

## Abstract

**Background and Objectives:**

This study examines interactions in residential care for people with dementia and severe challenging behavior. Approaching dementia as contextualized, lived experience, it explores how meaningful encounters can be fostered for residents and those directly involved in their life and care.

**Research Design and Methods:**

A phenomenological design informed by Todres’ embodied enquiry was employed. Data were collected through intensive shadowing observations (35 hr) over a 2-week period in a Dutch closed long-term care facility accommodating 16 residents. The daily work of 18 paid care workers was observed.

**Results:**

Meaningful interaction emerged as a delicate balancing process, described as *walking a tightrope*, requiring continuous attunement to both pleasant and distressing dimensions of lived experience. Care workers fostered at-homeness, togetherness, and shared memories, while also acknowledging disorientation, loneliness, grief, and sadness. This balancing act unfolded across four interconnected dimensions: (1) at-homeness versus uncanniness in space, (2) togetherness versus loneliness in relationships, (3) continuous recalibration in embodied attunement, and (4) joy versus sorrow in personal narratives.

**Discussion and Implications:**

While previous research has emphasized the importance of connectedness for meaning in life, this study underscores another crucial perspective: genuine well-being requires sincere engagement with life’s darker dimensions, not merely positive reassurance. Meaningful interaction depends on care workers’ capacity to remain present with both affirming and unsettling aspects of life. This calls for a relational approach to care that acknowledges distress alongside positive experience, supports agency through doing-with, and employs inclusive, non-corrective communication practices that flexibly integrate clinical and humanistic perspectives.

For all individuals, including those living with dementia, meaning in life is highly significant, and meaningful interactions play a vital role in well-being. Research has indicated that social engagement and experiencing oneself as being significant to others are crucial (Baumeister & Leary, 2017), and that people with dementia can continue to derive profound enjoyment from such interactions (Dewitte et al., 2019, 2021; Kloos et al., 2024). However, these sources of meaning are placed under considerable strain by the profound challenges associated with dementia, which are marked by multiple forms of loss. At the personal level, individuals may experience cognitive decline, loss of abilities (including self-governance and emotional regulation), reduced autonomy, and, in some cases, a diminished sense of identity. At the social level, dementia reshapes roles and relationships, often complicating the maintenance of meaningful connections and increasing the risk of social isolation. These challenges are further exacerbated by persistent negative stereotypes prevalent in both public and professional discourse (Nguyen & Li, 2020).

Together, the losses associated with dementia can profoundly affect the quality of relationships and interactions, thereby undermining overall well-being. Although individuals with dementia generally continue to long for companionship and connection, they may experience an increasing sense of distance from significant others (Dewitte et al., 2021; van Wijngaarden et al., 2019). While seeking a safe and accepting environment, they frequently encounter the disapproving or inquisitive gaze of others, whether from society at large or from close relatives (van Wijngaarden et al., 2019).

These relational difficulties are further intensified when individuals with dementia exhibit challenging behavior, such as agitation, verbal or physical aggression, repetitive vocalizations, disinhibition, or sleep–wake disturbances (Gerritsen et al., 2019). Such behaviors are highly prevalent in nursing home settings, affecting approximately 80% of residents (Selbæk et al., 2013). Within this population, a smaller but significant group displays very severe or extreme behaviors, including severe agitation (6.3%) and very frequent agitation (7.4%) (Palm et al., 2018; Veldwijk-Rouwenhorst et al., 2017).

Severe challenging behavior, particularly when agitation and aggression are frequent, persistent, and unpredictable, has far-reaching consequences. It is strongly associated with reduced well-being of residents and heightened distress, burden, and strain for both care workers and family caregivers (van Voorden et al., 2023). In some cases, the intensity of these behaviors exceeds the capacity of regular long-term care facilities, necessitating admission to specialized or psychiatric care settings (van Voorden et al., 2025). In the Netherlands, highly specialized units have been developed to provide diagnostic assessment and treatment for individuals with dementia and very severe challenging behavior. These units offer care when continued residence in regular dementia special care units is no longer feasible (Koopmans et al., 2022; van Voorden et al., 2023; Verhees et al., 2024).

There is broad consensus that people receiving care should be involved as fully as possible in decisions and practices affecting their daily lives, regardless of the severity of cognitive or behavioral impairments. In dementia care, this principle has informed a “doing-with” approach, which emphasizes residents’ active participation in everyday life and care practices (Novy et al., 2023). From a human rights perspective, it is further argued that people who communicate differently should be enabled to do so on equal terms and from their own experiential standpoint (Brannelly, 2011; Clare & Cox, 2003; Novy et al., 2023). Together, these perspectives underscore the importance of understanding which types of interaction facilitate more equitable and meaningful communication during care activities, especially when communication becomes more complicated due to impairments (Novy et al., 2023; Webb, 2017).

In recent work, van Voorden et al. (2023) have begun to map conditions for successful care in contexts of severe challenging behavior, and found that experts consider multidisciplinary assessment, person-centered approaches, and a skilled, well-functioning team essential for effective treatment of people with dementia and severe challenging behavior (van Voorden et al., 2023). Key elements included consistency, an understanding of the meaning of behaviors, appropriate responses, and an open attitude (van Voorden et al., 2023). Importantly, treatment success was defined not only in terms of improved well-being of the person with dementia, but also in terms of benefits for care workers, relatives, and fellow residents, reflecting a relationship-centered rather than a solely person-centered model of care (Nolan et al., 2004; van Voorden et al., 2023).

While these findings highlight the importance of attunement to people with dementia and severe challenging behavior, they are largely based on expert perspectives. To understand how meaningful interaction is actually realized in residential care, it is essential to examine what occurs in everyday encounters between residents and those involved in their care. As Novy et al. (2023) argue, the critical issue is not merely *whether* interaction takes place, but *how* it unfolds and what renders it meaningful. This calls for close attention to embodied, lived experience and to the relational context in which interactions are embedded. However, their meta-ethnography demonstrates that most empirical studies examine relational dynamics from a single perspective—either that of care workers or of care recipients—while relatively few focus on the interactive processes emerging between them (Novy et al., 2023).

Specialized residential settings for people with severe challenging behavior, where interactions may escalate more rapidly and where communication is both highly visible and consequential, are particularly suited for conducting this type of research. Moreover, staff in these settings are typically well-trained and experienced. They offer valuable insight into interactional practices that may prevent or de-escalate challenging situations, with relevance for dementia care beyond specialized dementia units. To address this gap, the present study explores how meaningful interaction is fostered between people with dementia and severe challenging behavior and those directly involved in their care in long-term care facilities. The guiding research question is: *How does meaningful communication and interaction between people with dementia and the care workers occur, and what makes these encounters meaningful?*

## Method

### Study design

This study employed a phenomenological research design informed by Todres’ approach to embodied enquiry (Todres, 2007, 2008). Embodied enquiry emphasizes the active use of the researcher’s body in data collection and analysis, as well as the creation of resonance in the reader, thereby enabling them to imaginatively enter the phenomenon. Intensive observations, known as “shadowing,” served as the primary data collection method. This observational approach involves observing individuals or a practice over an extended period (van der Meide et al., 2013, 2015). Unlike participatory observation, in which the researcher adopts multiple roles, shadowing involves remaining “as a shadow”: present in the situation but primarily focused on observing and understanding from the participants’ perspective. In shadowing, both the individual perspective and the surrounding context in which experiences occur are considered. As such, the method facilitates an embodied and contextualized understanding of the phenomenon, and enables the researcher to be present as the phenomenon unfolds (Todres, 2007). Paying close attention to the sensory expressions of participants, such as gestures, facial expressions, and posture, as well as to bodily responses that may be evoked in the researcher, can provide insights that go beyond verbal accounts. Moreover, while language is a key vehicle of meaning, some experiences are more readily perceived in embodied expression than articulated in words. This attention to meaning expressed through the body, combined with the prolonged presence of the researcher, renders shadowing particularly suitable for exploring the experiences of people with limited cognitive or verbal capacities, such as those with advanced dementia.

### Context

The study was conducted in a long-term care facility for older adults in the south of the Netherlands. Data were collected in a specialized closed unit for individuals with advanced dementia and very severe challenging behavior (Dutch: DZEP), which is staffed by specially trained nursing personnel and equipped with specific tools such as sensors, high-low beds, posey beds (a tent-like enclosure entirely covering a bed), bed rails, mobile lifts, and jumpsuits to support residents’ safety and comfort. The unit housed 16 residents, each with a private room and shared sanitary facilities. During the observation period, the first author (EW) observed 14 residents and 18 care workers. Most residents and care workers were observed on multiple occasions across different days.

The unit was purposively selected in consultation with a research coordinator and a unit manager from the care facility, as its high-intensity care context offers particular insight into interactional practices through which challenging situations may be prevented or de-escalated, and meaningful interaction may be fostered. Care workers were not individually selected; all staff members working on the unit were invited to participate and provided informed consent (see also Ethical Considerations).

The multidisciplinary team consisted of paid care staff with varied professional backgrounds and roles, including registered nurses, nursing assistants, and other support staff. Team members contributed to daily care, hospitality, and well-being activities, combining responsibilities related to physical care (e.g., personal hygiene and medication administration) with broader aspects of residents’ well-being (e.g., informal conversation, ball games, hair styling, and assistance with personal appearance such as nail care). Two care workers had specific responsibilities for hospitality in the living room and kitchen. One person was responsible for replenishing supplies, ranging from towels and bedding to cleaning cloths. Another professional, who referred to himself as a “jack of all trades,” regularly stopped by and could step in wherever needed, both in this unit and on others. He knew all the residents by name. In addition, a hairdresser, a dentist, a prevention worker, and a physiotherapist were observed at work (see Supplementary Appendix 1 for an overview of all professionals).

A daily schedule was in place that organized care around a shared group rhythm (e.g., set times for waking, washing, meals, coffee breaks, rest periods). This was, however, routinely adapted to residents’ individual needs and moods. During the observation period, variations in participation were accommodated smoothly without disrupting the overall flow of care, and alternatives (e.g., for meals) were arranged as needed. The number of staff present varied somewhat per shift. On average, there were approximately three professional care workers per shift, who were primarily responsible for nursing tasks. In addition, there was always at least one host responsible for the kitchen and living area, who was often supported in the mornings by a second host primarily providing individual support. During some shifts, an additional staff member was present to focus specifically on residents’ well-being. This meant that there were often around five professionals present on the ward. The other professionals typically joined the group more flexibly, sometimes by appointment, but often informally as needed. In total, the observed team consisted of 18 care workers. In addition, a unit manager who was not directly involved in daily care but who was personally familiar with the residents was regularly present and interacted informally with them (e.g., joining them for coffee).

### Data collection

The study encompassed approximately 35 hr of observation over a 2-week period. Following the instructions of Emerson et al. (2011), the researcher used an observation guide (see Supplementary Appendix 2). Observations were meticulously recorded in field notes made during and immediately after each session (Emerson et al., 2011). The researcher was physically present in daily care settings, employing all senses to observe and document interactions and experiences. Observations occurred during a range of activities, including personal care routines (e.g., helping residents out of bed, bathing, dressing), medication administration, social moments in the shared living spaces (e.g., coffee breaks, communal meals), and specialized visits (e.g., hairdresser, dentist).

In addition to observations, short, informal conversations were conducted with both residents and care staff. These conversations were not guided by a pre-structured interview schedule and occurred spontaneously during routine activities or in moments of quiet engagement. These exchanges served several purposes: sometimes to better understand the experiences and identities of the residents, or to clarify or contextualize observed behaviors and interactions; other times to foster trust or to nurture a comfortable atmosphere. Additionally, some anonymized photos of the care facility and its interior were taken to capture the ambience of the setting.

The empirical material consisted of detailed field notes documenting the observations and informal conversations during the researcher’s stay in the unit, supplemented by a reflective diary in which the researcher recorded emotional responses, reflexive insights, and preliminary interpretations.

### Data analysis

Data analysis followed the reflective lifeworld approach (Dahlberg et al., 2008). The analytic process began with immersion in the material through repeated readings of the field notes. Observational accounts, descriptive material, and reflexive entries were subsequently integrated into rich narrative descriptions addressing different dimensions of the research question. These narrative descriptions were iteratively re-read to identify smaller meaningful parts within the descriptions while maintaining proximity to the original textual material. These small segments of meaning were then grouped into so-called “meaning units.” By putting together meaningful units that seemed to belong together, an evolving pattern of meaning was discovered. Subsequently, all clusters were related to each other through synthesis, resulting in a description of the overarching theme as well as its constituents (Dahlberg et al., 2008). Excel was used to organize and structure the coding and to develop themes.

Validation and refinement of these emergent themes occurred through two rounds of in-depth dialogue, organized and facilitated by the first author. The first involved three purposively selected representatives of the care staff (the unit manager and two previously observed nurses). The second included a broader group consisting of seven health professionals from the organization together with one family member, who had been recruited through an internal call. Both sessions were structured around case-based discussions and iterative reflection on emerging themes. Following these exchanges, the themes were further abstracted and consolidated through a hermeneutic process of oscillation between concrete descriptions and more abstract thematizations. This iterative movement yielded a coherent structure of meaning and its constituent elements (Dahlberg et al., 2008).

The reporting was carried out in accordance with the COnsolidated criteria for REporting Qualitative research (COREQ) (Tong et al., 2007) (see Supplementary Appendix 3).

### Positionality

The first author leads a research group that focuses on ethical and existential issues in the last phase of life. She has extensive experience as a healthcare chaplain and ethicist and has worked across a range of healthcare settings, including dementia care. The second author is trained as a geriatric psychologist and leads a regional academic network in the field of long-term care for older adults and individuals with chronic illness. The objective of this network is to develop, disseminate, and implement scientific knowledge in both healthcare practice and education.

### Ethical considerations

The local Ethics Review Committee Oost-Nederland, after reviewing the protocol (no. 2022-16100), declared the study was not subject to the Medical Research Involving Human Subjects Act. The study was conducted in accordance with Dutch law and the Declaration of Helsinki. Ethical principles, including privacy and confidentiality, were upheld throughout the research process. Consent was obtained from the facility’s leadership, nursing team representatives, and the legal representatives of 14 residents. Observations excluded the two residents whose legal representatives did not provide consent.

Throughout the study, the researcher adopted a reflexive and ethically attuned stance in order to safeguard the integrity of all participants. This was achieved by agreeing that, in addition to obtaining general informed consent, each observational moment would be assessed individually to ensure ongoing verbal or non-verbal consent or assent from the resident. Particular care was taken during more intimate or personal activities (such as bathing and dressing), which were approached with heightened sensitivity. These situations were carefully aligned with the care staff’s prior experiences with each resident (e.g., whether the resident was comfortable with the presence of others, such as students or trainees, or whether this might cause distress). The researcher and the care worker also established in advance that, should any—even minimal—signs of discomfort or agitation arise, the researcher would immediately withdraw from the situation and discuss the circumstances afterwards with the care worker.

Ensuring anonymization, particularly for external parties, was a priority during the writing of this paper. However, this is also a complex task. Saunders et al. (2015) offer valuable strategies for anonymization, such as disguising names and locations. At the same time, they also warn against excessive anonymization that could compromise the integrity or meaning of the data (Saunders et al., 2015). In this paper, the aim is to find a balance between two important priorities: protecting participant identity and preserving the authenticity and contextual richness of the data. This is why the residents’ names have been altered, all female care workers are referred to as “Debora,” and male staff members are named “Benno.”

## Results

Meaningful interaction between care workers and individuals with dementia who at times exhibit severely challenging behavior can be conceptualized as walking a tightrope, that is, a delicate balancing act, requiring constant sensitive navigation between—and accommodation of—both pleasant and unpleasant aspects of the lived experience of dementia. On the one hand, it involves the deliberate effort to foster a sense of at-homeness, togetherness, and the preservation of happy memories. On the other hand, it demands an openness to recognizing and validating emotions such as disorientation, loneliness, grief, and sadness. The following four constituents delineate distinct facets of this intricate balancing endeavor, illustrating how care workers thoughtfully adjust and navigate shifting spheres and moods: (1) the duality of at-homeness and uncanniness in space; (2) the tension of togetherness and loneliness in relationships; (3) the continuous recalibration in embodied attunement, and (4) the interplay between joy and sorrow in personal narratives. Each constituent is described below and exemplified through real-life case illustrations as observed during the field visits.

### The duality of at-homeness and uncanniness in space

The closed dementia care unit was intentionally and carefully designed to cultivate a sense of belonging and coziness. Staff invested considerable effort in shaping an environment that feels both homelike and comforting, one in which residents, as well as staff members themselves, could feel at ease. Particular attention seemed to be directed towards fostering immediate and tangible forms of connection within the unit. These included personal greetings at the reception desk, the use of first names and local dialects, the sharing of homemade treats such as Valentine’s Day cake, and the continuation of communal traditions, including carnival celebrations. Together, these practices contributed to the creation of a familial atmosphere. Small, personalized gestures of care (e.g., neatly styled hair, manicured nails, a subtle touch of perfume, or thoughtfully chosen clothing complemented by matching accessories) served to further reinforce this environment by signaling recognition and dignity for each individual. Residents appeared to respond positively to these efforts, as reflected in mutual eye contact, smiles, and reciprocal expressions of appreciation.

Yet beneath this carefully cultivated homelike atmosphere, a latent sense of enclosure persisted within the unit. This became evident in several ways. The care facility, with its long corridors and recurrent locked doors, bore the characteristics of an institutional maze. The hallways, often marked by silence, were punctuated by warning signs reminding staff and visitors that residents were not permitted to leave the building. While these measures were, of course, intended to ensure residents’ safety, they simultaneously underscored—both for the researcher as a visitor and, presumably, for residents and other visitors alike—the boundary separating the care facility from the outside world. A similar ambivalence was reflected in the presence of a regional newspaper. On the one hand, it appeared to function as an attempt to bring elements of the outside world and everyday life into the unit. On the other hand, its largely untouched presence on the living room table symbolized, particularly from the researcher’s perspective as a visitor, the distance from life beyond the institutional setting.

At times, the carefully cultivated sense of homeliness stood in marked tension with more unsettling aspects of daily life in the unit. Sudden outbursts of crying or the sight of residents wandering aimlessly through the corridors contributed to an atmosphere that could be described as somewhat uncanny. As residents frequently experienced disorientation in time, place, and person, and consequently a diminished sense of control, they often appeared to engage in what might be understood as support-seeking behavior. This manifested, for instance, in repeatedly asking where they were or when they would be able to go home, seemingly as a means of coping with feelings of uncertainty or anxiety. Staff members generally responded by attempting to normalize the situation: offering reassurance, adapting to residents’ frames of reference rather than correcting them, and using physical proximity or gentle touch to convey comfort and calm, as illustrated in Box 1.

Box 1 Willem

Willem has very dark eyes, and he often looks at you with an intense, serious gaze, as if he’s looking straight through you. Occasionally, his expression softens, appearing a bit more cheerful. Willem hardly ever speaks, but he frequently makes an unintelligible sound. Then, suddenly, he stands up and walks with a determined stride straight towards something; the kitchen, the hallway. On the one hand, he seems to move directly towards his goal, but once he reaches the kitchen or hallway, he looks around restlessly and aimlessly, as if unsure how he ended up there.

At one point, I am walking in the hallway and come across Willem and Benno. Willem extends his hand towards me. Benno says, ‘Take his hand and walk with us for a bit.’ I take Willem’s hand and feel how tightly he clasps mine. Together, the three of us walk down the hallway towards Willem’s room, where we end up sitting together on his bed. ‘This is something we do often,’ Benno explains. ‘He’s restless now. By reaching out to him and offering your hand, you make contact, and it helps him to gradually calm down.’

A similar dynamic was observed when residents expressed a desire to leave the unit. It was not uncommon to see individuals pacing back and forth near the exit, hovering around the locked door as if waiting for an opportunity to leave. When another person needed to exit the unit, staff would typically approach the resident, gently linking arms and subtly guiding them in the opposite direction, without applying force. This allowed the departing person to open the door, step outside, and close it behind them without the resident leaving the unit as well. When residents nevertheless attempted to follow, staff repeated the process—patiently and without visible frustration—as many times as necessary.

While some residents appeared to contribute, probably unconsciously, to the unsettling atmosphere of the unit, without explicitly recognizing it as such, others seemed more aware of the ambivalence inherent in their surroundings. These residents conveyed mixed signals, sometimes even within a single moment: on the one hand expressing enjoyment of the convivial atmosphere and social interaction, and on the other articulating discomfort or a lack of feeling at home. Bart, for example, was one of the residents who spent much of his time in the shared living room. He enjoyed sitting at the large dining table, greeting everyone who entered, drinking coffee, and engaging in conversation. Yet his engagement could swiftly shift. At times, he became visibly irritated by the behavior of fellow residents. During the researcher’s visits, he frequently commented—sometimes quite sharply—on another resident who paced restlessly through the room. “*Oh, stop it!*” he would exclaim. On other occasions, he sought confirmation of his irritation from others, including staff members or the researcher: “*There he goes again. He never sits still. Got ants in his pants*.” At times, his remarks were tinged with a sense of weary resignation, for instance when he sighed: “*This place really is a madhouse*.”

Within the microcosm of the unit, interactions between staff and residents thus unfolded in a space characterized by a lingering duality: a space that offered comfort, familiarity, and belonging, while simultaneously carrying an undercurrent of unease, disorientation, and confinement. Explicitly acknowledging this coexistence of at-homeness and uncanniness—particularly for residents who expressed such ambivalence—seemed crucial for fostering meaningful and responsive interaction. This involved, for example, care workers recognizing when a resident did not feel fully at home or felt misplaced, and not disregarding these experiences.

### The tension of togetherness and loneliness in relationships

While the first constituent is more related to the spatial environment in which people lived, there appeared to be somewhat similar tension on the relational level. It concerned a poignant tension between fostering a sense of togetherness and addressing a pervasive feeling of loneliness that many residents seemed to experience. On the one hand, care workers strived to create a sense of “we,” a shared feeling of connection, often between a resident and a care worker. This sense of togetherness between a resident and a care worker was cultivated through eye contact, engaging in emphatic touch, deliberately thanking residents during care moments for the joint effort, and addressing them warmly by name, often using familiar expressions from the local dialect, such as the typically southern Dutch forms “*ons* *Bart*” (“our Bart”) or “*ons* *Tineke*” (“our Tineke”). Although to a lesser extent, there were moments that they also tried to create this feeling among residents as a group. Activities such as shared games, using objects like a ball, or a visit from the therapy dog, created small moments of interaction and joy that appeared to be joyful and meaningful not only for residents, but also for care workers.

At the same time, in spite of these efforts, a profound emotional loneliness often persisted among the residents. This became particularly acute in cases where residents experienced a mismatch between themselves and the group dynamic, such as when a resident retained cognitive awareness but felt out of place among those further along in their dementia journey. It demonstrated a dual reality of being physically present among others but emotionally isolated. Despite the communal setting, the inner world of many residents seemed to remain deeply private, unshared, and often unacknowledged. Acknowledging this dual sense of togetherness and loneliness may facilitate residents’ experience of being recognized and affirmed.

Franca’s story, in Box 2, illustrates that, while caregiving practices often aim to foster connection, such efforts may fall short for residents who feel that their cognitive, emotional, or biographical trajectory does not align with those of their fellow residents. For Franca, the shared environment does not translate into a sense of belonging; rather, it seems to intensify feelings of frustration and isolation. Her experience highlights the limits of collective approaches to togetherness and points to the importance of remaining attentive to individual forms of loneliness that can persist even within ostensibly social and caring environments.

Box 2 Franca

Franca feels different; she emphasises that she does not have dementia. Debora tells me that she actually has a less common form of dementia, a so-called frontotemporal dementia. One morning while she is being cared for, Debora says to me, ‘You’d better wait in the hallway. I don’t think she’d appreciate you coming along.’ Franca speaks loudly and sharply: ‘Hold on, take it easy! Wait a minute!’ Later, Debora explains, ‘She’s very direct. She used to run a taxi company, and it was clear who was in charge there. She is still good at giving us orders. And as far as I’m concerned, her frustration is understandable.’

Franca likes eating with the group because she enjoys being around people. However, her presence is marked by angry and disinhibited behaviour. Every afternoon, precisely ten minutes before mealtime, she arrives with her walker. She heads straight to the small square table by the window, facing outward, where she can sit alone, her back to the kitchen and the room, gazing outside. As soon as she sits down, she wants to be pushed closer to the table. Without turning around, she calls out sharply and curtly, ‘Nurse, can you push me in now?’ She also gives orders about the food: ‘Nurse, a half portion!’ ‘No raw vegetables; I never want those!’ and ‘Where’s the napkin!’

On the final day of my visit, Franca briefly gives me a stern look as she enters the living room. I start a conversation by introducing myself and explaining what I’m doing there. She looks at me sceptically and says: ‘Research? And what can we expect from that?’ I briefly explain the purpose of the research and ask her how she feels about living in this unit. That seems to break the ice. She talks at length about her experiences: that she would prefer to watch television in the living room, instead of alone in her room. Or that she enjoys listening to music together with others. She’s frustrated that this is often ‘too much’ or ‘too overwhelming’ for the others, and that consequently she is frequently told ‘no’.

‘The care is fine, even good. The food is good. But all those rules…, rules this, rules that…’ She also had a hard time adjusting. For example, she tells me she was used to having soup before the main meal. ‘Here, you get your main meal straight away and then soup later in the evening with bread.’

Despite her dissatisfaction, Franca doesn’t want to move again. ‘I want to die here. It would actually be fine if I died. Nobody will miss me.’ She explains that she finds no companionship among the other residents and feels she can’t talk to anyone. ‘You can’t have a meaningful conversation with them anymore.’ Once a month, she has a chat with the health chaplain. Beyond that, she feels she has little interaction. I question whether, through her frustration and irritability, she may be creating additional distance between herself and the others.

### Continuous recalibration in embodied attunement

With great sensitivity, care workers continuously sought to attune themselves to both the emotional states of individual residents and the overall atmosphere of the group. This required a receptive attentiveness to subtle, often non-verbal cues. Care workers adjusted their movements, tone of voice, and pace in response to residents’ expressions. Without romanticizing it, at times it resembled a form of embodied synchrony—a joint dance—in which they sought to align their own presence with the inner world of the person with dementia. Strategies of attunement included gentle, mindful touch, sustained eye contact, patience, and the incorporation of elements of residents’ personal histories and identities into everyday interactions. This embodied form of care was far from straightforward. Attunement demanded continuous recalibration: care workers had to carefully navigate the tension between providing stimulation and avoiding overstimulation, while simultaneously remaining responsive to the diversity of residents’ needs.

Joyce’s story, in Box 3, shows that embodied attunement often entailed a double balancing act. On the one hand, they had to attune to the unpredictable rhythms, emotions, and behaviors of each individual resident. On the other, they had to maintain the overall atmosphere of the group and mitigate the potentially disruptive impact one person’s distress or agitation could have on others. Care workers constantly had to negotiate between multiple, sometimes competing, forms of responsiveness. Embodied attunement thus resembled walking a tightrope in that it involved the possibility of tilting too far to either side, and thus demanded sensitivity, precision, and ongoing adjustment within a dynamic context.

Box 3 Joyce

Joyce is sitting in the living room in one of the armchairs. She looks well-groomed and stylish. She is wearing beautiful jewellery and her nails are painted. When Debora-1 enters the living room, she walks over to her, kneels to be at eye level, places a hand on her knee, and greets her personally. ‘Good morning, Joyce. How are you today?’

The atmosphere in the living room is good and pleasant. There is laughter and conversation. People are making jokes. And the interaction with Joyce adds a lively buzz to the group. Joyce wants to dance, she talks about getting married, and winks at the some of the care staff. It is undoubtedly a joyful moment. Gradually, however, Joyce becomes more restless. With quick steps, she starts moving from chair to chair, stands up, and plops down in another armchair that is free, repeating this movement several times. Also, her gaze is changing, and tension is showing in the knuckles of her hands.

There is a brown-green stuffed dog made of soft fleece material in one of the armchairs. It is heavy, and when you press it, it moves slightly. It is regularly used to calm people down and is sometimes placed on their lap. Suddenly, Joyce jumps up from her chair, grabs the dog, and forcefully throws it onto the lap of one of the other residents. He is startled and lets out a short scream. Joyce then starts growling loudly and walks quickly to the hallway. You can hear her calling. Debora has already approached the disturbance. She moves smoothly behind Joyce, steps in firmly, walks a little in the same rhythm, grabs her gripping hand, rubs and strokes it, and then guides her in a smooth, straight motion to the calm of her own room. After closing the door behind her, the noise gradually fades. The atmosphere in the living room shifted noticeably, and there was a palpable tension among the residents who all remained seated in the circle during the commotion.

Afterwards, Debora tells me that it always presents her with a big dilemma: ‘You want her to enjoy herself, you know, and not take away every joyful moment in advance, cut everything short. But there’s always the risk that we let it go on just a little too long, that it tips over. The trick is to pull her out just in time. Most of the time, we see it, but today, I was a little too late, and then her tension builds up too much. And it tips over. Then we have to help her calm down again, in her own room, in the posey bed and with some oxazepam. But we mustn’t forget that beautiful moment either.’

The observed interactions further revealed that embodied attunement involves more than subtle tactile engagement and sustained visual connection; it also requires temporal sensitivity, unhurried presence, and quite a bit of waiting. Care workers often had to wait for the right moment, attuning themselves to the rhythm of the interaction rather than imposing direction or control. By sensing when to move and when to hold back, they were able to align with residents’ emotional states and, at times, pre-empt moments of escalation. Temporal sensitivity proved central to preventing tension from taking hold and to sustaining a more harmonious relational dynamic, as illustrated in Box 4.

Box 4 Pieter

We are doing the morning round and heading to Pieter. Benno is already warming some washcloths in the microwave. He says to me: ‘I’m going to check on Pieter. If he wants to get up, I need to seize the moment immediately, because his mood can change in an instant. It’s important that I don’t have to go and warm up the cloths at that point. But it could also be that he doesn’t want to get up at all. Forcing it would be pointless. In fact, it could escalate and even become physical.’

Softly, Benno enters the room. It’s still dark, and he leaves it that way. He leans over the bed and gently says, ‘Good morning, Pieter. Would you like to get up?’ Pieter responds very clearly: ‘Please, no!’ Benno replies, ‘All right, Pieter. See you later.’ Out in the hallway, Benno says to me: ‘We only do it when he’s ready. Even if it’s in the afternoon. And even if I have to warm those washcloths five times.’

I realise that I’ve only seen Pieter out of bed a few times in the low-stimulus area of the living room. Most of the time, he stays in his bed. When he does sit in the living room, he’s often alone at a small table, eating or drinking something. Once he’s had enough, he starts making gurgling sounds and spits next to his wheelchair; a clear sign for the staff that he wants to return to his own room.

On the final day of my visit, Pieter remained in bed throughout the morning. Benno checked on him repeatedly, but he was resolute in his refusal to get up. But shortly before I was about to say goodbye in the living room, mid-afternoon, a colleague suddenly wheeled Pieter inside. His face lit up with a broad smile, and his eyes appeared unusually clear and bright. The room responded immediately: those present expressed genuine delight at seeing him so animated. Almost instinctively, they formed an improvised honour guard as he entered. Pieter laughed and lifted his arms in triumph, as if celebrating a marathon victory. It was a brief but meaningful moment of collective joy, and one that was exceptionally rare for Pieter.

A different dimension of this continual recalibration in attunement also became apparent. During episodes of severe behavioral disturbance or heightened stress, care workers tended to shift from an embodied mode of engagement to a more observational and diagnostic stance, particularly when discussing the situation among themselves or with the researcher. In such moments, residents’ behaviors were often interpreted through psychiatric or medical frameworks. Reflecting with the care workers on these shifting postures suggested that this diagnostic framing partly served to externalize the cause of an incident, locating it outside the immediate interaction, either in contextual conditions or in the resident’s underlying pathology. This reframing appeared to offer a sense of reassurance in situations that might otherwise be experienced as an inability to respond effectively. At the same time, understanding challenging behavior as intentional—for instance, as an attempt to manage uncertainty or anxiety—also tended to enable a more empathetic orientation towards residents.

This tension became evident during an encounter with Simone. Before entering her room, Debora mentioned that Simone often resists care and prefers to stay in bed. When we entered, the room was still dark and had a musty smell. Debora approached the bed and softly called Simone’s name. After a brief pause, Simone suddenly looked up, visibly frightened, and began to speak loudly and urgently, telling a very confused and fragmented story about people being beaten and gunshots. She kept on repeating that she had been calling for help and that no one had come. Her distress escalated quickly, her voice rising as she accused Debora of ignoring her. Rather than correcting or contradicting her, Debora stayed close, crouching beside the bed, maintaining eye contact, and stroking her arm while listening without interrupting. When Simone stopped talking, Debora responded calmly, apologizing for not having heard her earlier, acknowledging her distress, and reassuring her of her presence. Later, outside the room, Debora reflected on the encounter. She explained that such episodes occurred more frequently, that residents, including Simone, sometimes see or hear things that are not there. The team had been trying to figure out if this could be related to trauma or another underlying condition. In this moment, the mode of engagement subtly shifted: what had begun as an embodied, relational effort to remain with Simone in her distress now changed into a different way of relating to the situation, namely a clinical-diagnostic reflection on symptoms and possible explanations.

This episode illustrates how caregiving continually moves between different modes of understanding. On the one hand, care workers strived to remain attuned to residents’ immediate experiences, responding through presence, touch, and calm reassurance. On the other, they also wish to interpret and contain behaviors that are confusing, distressing, or disruptive by drawing on professional frameworks and pathologizing jargon. The latter tended to frame problematic behavior as the consequence of personal dysfunction, whereas the former may emphasize the mismatch in the bi-directional interaction between care worker and resident. The oscillation between these modes—between embodied responsiveness and diagnostic reasoning—reveals the fragile balance at the heart of everyday care, where meaning-making, emotional labor, and clinical judgment are constantly interwoven.

### The interplay between joy and sorrow in personal narratives

At the unit, care workers faced a profound tension between honoring the personal narratives of residents and grappling with the sadness and loss that are often inherently embedded in the stories of their residents. On the one hand, they put effort into becoming familiar with residents’ life histories as a means to honor their identity and preserve these personal narratives as well as their dignity. Such narrative attunement involved acknowledging residents as whole individuals, with names, histories, and accomplishments that transcend their current condition. Care workers tried to bring personal stories to life by practices such as referencing significant life events and incorporating personal belongings such as photos, furniture, or cherished mementos into the care environment. Residents’ voices and their life histories, even when fading, could be restored through the storytelling of care workers or family members. As such, they tried to foster a sense of continuity between residents’ past and present selves and create a space where the resident’s former identity and achievements were celebrated, which helped to counterbalance feelings of loss, shame, or uselessness.

However, despite the importance of narrative attunement, the care workers often struggled to confront the sadness, trauma, or sense of loss inherent in the stories of those they cared for. This tension between preserving identity and recognizing loss often became visible in the care environment. For instance, a photo of a former sailboat or sports car could evoke a sense of happiness, freedom, and perhaps even pride in the resident, but it simultaneously contrasted starkly with the constraints of a closed unit or a posey bed, which symbolized deep dependency and loss of autonomy.

Box 5 Alfons

Beaming, Alfons enters the room, and right behind him, his wife peeks her head around the door. She’s visiting today. They’re going for a walk together in the garden behind the house. Alfons has already put on his coat and cap. Enthusiastically, he says, 'I saw her coming through my window!' When his wife leaves an hour and a half later, his mood shifts, and he looks sad. 'Now I’m here again, alone.' One of the staff members says, 'But you’re never lonely here. We’re all here, and your dear wife will be back next week.' Alfons doesn’t reply, just shakes his head. A little while later he says to me, 'She said she had to go home, but that’s nonsense. There’s nobody there. She has all the time in the world. And still she had to leave so soon.' And then after a short silence: 'I won’t be able to leave this place ever again…'

It turned out to be quite difficult for most care workers to recognize and validate the sad aspects of life stories, as they expressed concern that residents might become overwhelmed. This discomfort could lead to a tendency to avoid or redirect attention away from expressions of loneliness, grief, or existential despair, as illustrated in Box 5. Although such responses may stem from a wish to uplift and distract, they seem to unintentionally dismiss the emotional reality of residents. This underscores the importance that care workers recognize both joy and sorrow simultaneously—without privileging one over the other—thereby integrating positive and painful aspects into a coherent fabric of interaction. It reflects the care worker’s task of making space for discordant emotions while fostering connection. In this sense, care may be understood as an ongoing act of preservation, repair, and appreciation of something fragile yet potentially deeply meaningful.

### The bidirectionality of meaningful care work

The care work itself, both in terms of team collaboration and engagement with people living with dementia, was experienced as highly meaningful by the staff. While on the one hand they described their approach as “perfectly normal,” it was simultaneously regarded as being of great value, also for themselves. Staff indicated that working in this way contributed to reducing and preventing challenging behaviors, and also de-escalating situations of tension or potential conflict, making for a more positive work experience. This highlights the bidirectional, reciprocal nature of meaningful care work: care workers were not only of significance to residents, but the work and the residents also gave meaning back to them. This was poignantly illustrated at the end of a shift, when a staff member responded to a colleague who asked, “*Are you going home?*” with: “*Yes, but actually this also feels like my home*.”

## Discussion

This observational study aimed to deepen our understanding of how meaningful forms of communication and interaction unfold in specialized residential settings for people with dementia and severely challenging behavior. The current study found that meaningful interaction between care workers and individuals with dementia involves a sensitive balancing act, metaphorically imagined as walking a tightrope. On a personal level, it requires embodied attunement, temporal sensitivity, and a constant, careful navigation of both pleasant and unpleasant emotional experiences. In order to make room for meaningful interactions, care workers should cultivate (micro-)moments of togetherness, of familiarity, belonging, and shared connection, while also accommodating and validating unsettling realities of disorientation, loneliness, grief, and loss in the unit. As such, they foster a space—literally and relationally—where people can feel at home and relate to others in a meaningful way. While responding sensitively to the fluctuating rhythms, emotions, and behaviors of individual residents, care workers are simultaneously required to safeguard the collective atmosphere of the group and to contain the potentially disruptive effects of one person’s distress on others. This entails a continuous process of negotiation between multiple, and at times competing, demands for responsiveness.

The present study corroborates earlier research demonstrating that meaningful attunement to people with dementia and the maintenance of genuine social relationships are central to dementia care (Dokos et al., 2023; Emilsson, 2008; Savundranayagam et al., 2016). By attending to residents’ life histories, values, and preferences, care workers sought to preserve identity and relational continuity. When care workers attune to residents’ individuality, interactions are more likely to be experienced as meaningful; conversely, directive or dismissive communication is associated with resistance, withdrawal, or distress (Dokos et al., 2023; Emilsson, 2008; Savundranayagam et al., 2016). Consistent with prior work (Dokos et al., 2023; Emilsson, 2008; Savundranayagam et al., 2016; Savundranayagam & Lee, 2017; Strandroos & Antelius, 2017), our findings further suggest that responsive relationships in dementia care are enacted through embodied and intersubjective practices. Care staff therefore draw on a range of embodied forms of attunement such as touch, timing, bodily presence, and tone of voice, to sustain and co-create meaningful interaction, particularly when verbal communication is limited.

Another key finding concerns the need to attend to the full breadth of residents’ experiences, including loss and sadness. While existing literature has largely focused on grief among family caregivers (Blandin & Pepin, 2017; Boss, 2010, 2011; Santulli & Blandin, 2015), our findings demonstrate that loss, loneliness, and sorrow are also integral to the lived experience of people with dementia, even when these cannot be articulated verbally. Therefore, meaningful care requires sincere engagement with distressing experiences rather than well-intentioned but minimizing or avoiding responses. Our study, however, also illustrates that in practice, care staff tend to find it difficult to relate to distressing experiences. Boss (2010) argues that this may reflect a broader cultural tendency within Western healthcare systems that privilege mastery, problem-solving, and closure, while leaving little room for ambiguity, helplessness, or unresolved suffering. Following Boss, among others, we argue that care practices may benefit from creating more space to acknowledge (and/or even ritualize) loss and sorrow, thereby supporting professionals, residents, and families in learning to live with unresolved suffering rather than prematurely redirecting these feelings (Boss, 2011; van Wijngaarden, 2021). As such, our findings underscore the importance of a lifeworld-oriented approach that embraces the full complexity of human experience in dementia care.

The findings of this study also reinforce the significance of understanding care as a reciprocal process, echoing earlier research conducted in the context of care for individuals with dementia (Brannelly, 2011; Brown Wilson & Davies, 2009; Novy et al., 2023; Watson, 2019). Caring is to be understood as a joint and responsive endeavor, regardless of how dependent or vulnerable residents may appear. It is about doing-with rather than doing-for. As Novy et al. (2023) emphasize, doing-for often reflects an underestimation of a resident’s ability to engage in care activities. This perspective places residents in a passive role and can result in a one-directional, task-driven approach, where those receiving care are treated as unable to contribute (Brannelly, 2011; Watson, 2019). Such relational imbalances are especially evident for residents who face communication challenges (Novy et al., 2023). In contrast, this study—like earlier work—illustrates that doing-with entails inclusivity, where care activities are approached as joint efforts. This requires attention to maintaining the resident’s agency wherever possible and recognizing care as a collaborative practice, expressed through small acts of mutual gratitude, supportive communication patterns, such as adopting inclusive “we-language,” following the resident’s lead, refraining from correcting their perceptions, and attentively awaiting a response (Brannelly, 2011; Kaufmann & Engel, 2016; Nolan et al., 2004; Novy et al., 2023; van Wijngaarden, 2025; Watson, 2019).

The findings of this study also support the broader shift in dementia research from a predominantly cognitive conception of self-focused on rational reflection and reasoning—towards a more multidimensional understanding of the self (van Woerkum-Rooker, 2025). In line with this perspective, the self is not merely reducible to reflective capacities but is also constituted through embodied experience, relational embeddedness, and continuous interaction with the environment (van Woerkum-Rooker, 2025). By illustrating how meaningful interactions unfold in residential dementia care, this study demonstrates that personhood and identity can be sustained through bodily attunement, shared presence, and social connection, even when cognitive and verbal expression decline. These insights underline the need for care practices that engage with the embodied and socially embedded dimensions of selfhood, thereby fostering a sense of meaning and well-being in advanced dementia (van Wijngaarden, 2025).

Finally, this study underscores the relevance of Martinsen’s distinction between a recording and a perceiving stance in dementia care (Martinsen, 2006, 2011). Whereas the recording stance is characterized by classification, assessment, and interpretation within biomedical frameworks, the perceiving stance emphasizes openness, emotional responsiveness, and attentiveness to the lived experience of the other. Our research shows that, in the context of dementia care, interpreting challenging behavior as a symptom of disease can foster greater empathy towards residents, provide insight into causes of certain behaviors, and provide clues for better aligned treatment. Our findings resonate with earlier work (Appleton & Pereira, 2019; Cohen-Mansfield et al., 2017; Emilsson, 2008; Gerritsen et al., 2019), highlighting the value of the recording stance, while simultaneously supporting Martinsen’s caution that relying too heavily on this perspective could lead to reducing individuals to diagnostic categories and obscuring their personhood. As our study demonstrates, good care depends on integrating both stances: the recording stance remains essential for clinical judgment and can help care workers to avoid personalizing or internalizing distressing behavior, while the perceiving stance enables embodied attunement, relational presence, and meaningful engagement. Holding these perspectives in tension appeared crucial for sustaining humane and responsive dementia care.

### Clinical implications

The findings of this study underscore that people with dementia, even if they display severe challenging behavior, remain active relational partners capable of contributing to meaningful interactions. Prior research indicates that care provision is constrained by emotional burden, challenges in dealing with challenging behaviors of dementia, and insufficient training (Dokos et al., 2023; Emilsson, 2008; Ericsson et al., 2013; Fetherstonhaugh et al., 2021; McGilton et al., 2012). Attuned communication—where care workers interpret behavioral expressions as communicative acts—fosters relational closeness and is associated with greater staff well-being and job satisfaction (Savundranayagam & Lee, 2017). The present study, conducted in a specialized unit, confirms these findings and provides valuable insights into interactional practices by showing how meaningful interactions are enacted. It also offers three concrete points for improving staff training, namely:

1. The study shows the importance of taking a lifeworld-oriented approach, which means engaging sincerely with both joys and sorrows. Well-being and meaning are supported not only by positive connectedness but also by explicitly recognizing sadness, loss, and despair. In order to sustain meaningful connections with people with advanced dementia, care workers should navigate a delicate balance between fostering togetherness and validating unsettling realities.
2. Care is understood as a joint, inclusive process—a reciprocal doing-with—in which residents are active participants rather than passive recipients, sustaining agency even in advanced stages of dementia by using inclusive language, temporal sensitivity, waiting, following the resident’s lead, and refraining from correction.
3. Meaningful care requires moving between a biomedical “recording” perspective and a receptive “perceiving” stance, balancing clinical and more humanistic stances.

### Strengths and limitations

This study offers unique insider perspectives through its embodied, phenomenological approach. The use of shadowing—a method rarely applied in this context—adds to the still limited body of qualitative research in the context of intensive dementia care, addressing a gap highlighted in the literature (Novy et al., 2023) and contributing to a growing understanding of the lived experience of dementia care (Novy et al., 2023). Several limitations should also be acknowledged. First, the study’s relatively short duration may have limited the extent to which longer-term processes, dynamics, or changes could be captured. Consequently, the findings may primarily reflect immediate interactions and experiences, rather than developments unfolding over time. However, this immediacy is particularly characteristic of advanced dementia care. This limitation is further balanced by the intensity of the observations, which encompassed a substantial number of residents, care workers and visiting relatives. Second, this study’s primary focus on interactions between care workers and residents within a closed care setting yielded valuable insights into everyday communication and relational dynamics. However, it is limited in its understanding of how broader contextual factors evolve and influence care practices over time. Future research could extend the scope of observations to better capture how the care environment, and the degree of autonomy or restriction it imposes, shapes residents’ sense of self and patterns of interaction.

## Supplementary material

Supplementary material is available at *The Gerontologist* online.

## Supplementary Material

gnag175_Supplementary_Data

## Data Availability

The data underlying this study contain sensitive personal information and cannot be shared publicly to protect participant privacy. Data access is therefore restricted in accordance with ethical and legal requirements. Qualified researchers may request limited access to the anonymized data by contacting the authors and upon approval by the relevant ethics board.

## Pre-registration

This study was not pre-registered.
