# Supplementary material for: Walking a tightrope: meaningful encounters in the care for people with dementia and severely challenging behavior
Source: Gerontologist. 2026 Aug 7;66(10):gnag175. doi: 10.1093/geront/gnag175 (PMC13623531; doi:10.1093/geront/gnag175)
Supplement: Supplementary file 1 [file gnag175_supplementary_data.docx]

**THREE SUPPLEMENTARY APPENDICES related to the article:**

***Walking a tightrope:*** *meaningful encounters in the care for people with dementia and severely challenging behaviour*

**SUPPLEMENTARY APPENDIX 1**

**Overview of observed care professionals**

| **Profession** | **Male** | **Female** |
| --- | --- | --- |
| Specialised nurse | 1 | 1 |
| Nurse | 1 | 6 |
| Care assistant (wellbeing) |  | 1 |
| Hospitality worker |  | 2 |
| Supplies assistant |  | 1 |
| All-round assistant (“jack of all trades”) | 1 |  |
| Hairdresser |  | 1 |
| Prevention worker | 1 |  |
| Physiotherapist | 1 |  |
| Dentist | 1 |  |
| Unit manager |  | 1 |

**SUPPLEMENTARY APPENDIX 2**

**The observation guide**

**Research question:** *How does meaningful communication and interaction between people with dementia and their professional care professionals occur, and what makes these encounters meaningful?*

**Main focus of the observations:** interactions between professional care professionals and residents

**Points of attention during the observations** (based on Emerson et al., 2011):

1. Initial impressions:
a. Key components of the observed interaction
b. (Detailed) sensory information about
i. Physical setting
1. Positioning of participants
2. Furniture/design of the spaces
3. Striking items and/or attributes
4. Sounds/colours/temperature/smell
ii. Involved participants
1. Appearance
2. Striking features
3. Posture/attitude
4. Impression
2. How are routine actions in the setting organized / take place?
3. What is/seems significant or unexpected?
a. Incidents
b. Emotions
c. Impressions
d. (non)verbal interactions
4. What is/seems to be significant or important to the participants?
a. When do they stop?
b. What do they talk about?
c. What produces (strong) emotional responses?
5. Other broad impressions (with help of the lifeworld dimensions; Todres, 2007)
a. Temporality
b. Spatiality
c. Intersubjectivity
d. Embodiment
e. Mood

**SUPPLEMENTARY APPENDIX 3**

**A COREQ-table; additional information on reporting according to the COnsolidated criteria for REporting Qualitative research** (Tong et al., 2007).

| **Reporting criteria** |  | |
| --- | --- | --- |
| **Domain 1: Research team and reflexivity** | | |
| Personal characteristics |  |  |
| Observer/researcher | Els van Wijngaarden | |
| Credentials | PhD | |
| Occupation | Associate Professor | |
| Gender | Female | |
| Experience and training | EW has carried out as well as supervised many qualitative research projects in diverse healthcare settings. Before her academic career, EW was a health chaplain and ethicist, trained and specialized in ethical and existential issues in the context of healthcare, including the care for people with dementia. | |
| Relationship with participants |  |  |
| Relationship established | Prior to the observation period, relationship was established with a program and research manager, a team leader and two nurses all related to the unit where the research project was planned. | |
| Participant knowledge of interviewer | All health professionals in the group knew the research interests and goals prior to interview. A short accessible version of the project was shared. Also at the start of each research day, the researcher introduced herself (and her project) to those the working that day. | |
| Interviewer characteristics | No further characteristics were reported. | |
| **Domain 2: Study design** | | |
| Theoretical framework |  |  |
| Methodological orientation and theory | This study employed a phenomenological research design informed by Todres’ approach to embodied enquiry (Todres, 2007, 2008). For data analysis, the study followed the process as described in the reflective lifeworld approach (Dahlberg et al., 2008). | |
| Participant selection |  |  |
| Sampling | Purposive sampling; The unit was purposively selected in consultation with a research coordinator and a team leader from the care facility (see article for further detail). | |
| Method of approach | Participants were approached personally by local contact persons. | |
| Sample size | One unit, housing 16 residents, During the observation period, also 18 care professionals were observed. | |
| Non-participation | Observations excluded the two residents whose legal representatives did not provide consent. No reason was provided. They just did not react to the contact person. | |
| Setting |  |  |
| Setting of data collection | Care facility in the south of the Netherlands, linked to the University Knowledge Network for Older Adult Care Nijmegen. | |
| Presence of non-participants | A few times, relatives were visiting the unit. They were already informed beforehand by email that this research project took place. They were not observed for research reasons. No one reported having reservations regarding the presence of a researcher. On the contrary, participants mainly expressed that they appreciated that research was being conducted specifically on this group. | |
| Description of sample | The multidisciplinary team comprised staff with specialized roles in daily care, hospitality, and well-being, alternating between tasks focused on physical care (e.g., personal hygiene and medication administration) and activities aimed at residents’ well-being (e.g., informal conversation, ball game, hair grooming, and assistance with personal appearance). One well-being supervisor focused entirely on providing individual guidance in the low stimulus living room. Two care professionals had specific responsibilities for hospitality in the living room and kitchen. One person was specifically responsible for replenishing supplies, ranging from towels and bedding to cleaning cloths. Another individual was referred to as a ‘jack of all trades’. In addition, a hairdresser, a dentist, a prevention worker, and a physiotherapist were observed. | |
| Data collection |  |  |
| Interview guide | The researcher created an exploratory observation guide for conducting her fieldwork (see Appendix 1). The focus was on observing (meaningful) interactions between care professionals and care recipients/residents.  There was no interview guide, as the interviews were purposively characterised as informal conversations. These conversations were not guided by a pre-structured interview schedule and occurred spontaneously during routine activities or in moments of quiet engagement. The interview guide was not piloted. | |
| Repeat interviews | No interviews were repeated. | |
| Audio/visual recording | No audio recording. | |
| Field notes | EW made field notes during the observations and immediately after. | |
| Duration | The duration of the observations was a whole working day, around 8 hours each day. | |
| Data saturation | Data saturation was not discussed. However, it is expected that sufficient data was collected due to the number of participants as well as the themes revealed in the analysis. | |
| Transcripts returned | No transcripts were returned to participants for comments. | |
| **Domain 3: Analysis and findings** |  |  |
| Data analysis |  |  |
| Number of data coders | One data coder (EW) | |
| Description of coding tree | The coding process was done following the reflective lifeworld approach method, as described in the paper. | |
| Derivation of themes | Themes were inductively derived from the collected data. | |
| Software | Excel | |
| Participant checking | Three representatives of the care staff (a team leader and two nurses all participating in the study) did provide feedback on the preliminary findings, as well as a broader group consisting of seven health professionals from the organization together with one family member. | |
| Reporting |  |  |
| Quotations presented | Quotations and detailed description of situations are presented throughout the paper in order to illustrate the findings. | |
| Data and findings consistent | There is consistency between the data presented in the paper and the findings. | |
| Clarity of major themes | Major themes are presented as clear as possible in the paper. | |
| Clarity of minor themes | Subthemes are also described as clear as possible. | |
